# An Intelligent Sensor Array Distributed System for Vibration Analysis and Acoustic Noise Characterization of a Linear Switched Reluctance Actuator

**DOI:** 10.3390/s120607614

**Published:** 2012-06-07

**Authors:** José Salvado, António Espírito-Santo, Maria Calado

**Affiliations:** 1 EST, UTCEI, Polytechnic Institute of Castelo Branco, Av. do Empresário, S/N, Castelo Branco 6000-767, Portugal; 2 FE, DEM, University of Beira Interior, Calçada Fonte do Lameiro, 1, Covilhã 6200-001, Portugal; E-Mail: rc@ubi.pt

**Keywords:** acoustic noise characterization, intelligent sensor array, microcontroller distributed system, vibrations monitoring, linear switched reluctance actuator

## Abstract

This paper proposes a distributed system for analysis and monitoring (DSAM) of vibrations and acoustic noise, which consists of an array of intelligent modules, sensor modules, communication bus and a host PC acting as data center. The main advantages of the DSAM are its modularity, scalability, and flexibility for use of different type of sensors/transducers, with analog or digital outputs, and for signals of different nature. Its final cost is also significantly lower than other available commercial solutions. The system is reconfigurable, can operate either with synchronous or asynchronous modes, with programmable sampling frequencies, 8-bit or 12-bit resolution and a memory buffer of 15 kbyte. It allows real-time data-acquisition for signals of different nature, in applications that require a large number of sensors, thus it is suited for monitoring of vibrations in Linear Switched Reluctance Actuators (LSRAs). The acquired data allows the full characterization of the LSRA in terms of its response to vibrations of structural origins, and the vibrations and acoustic noise emitted under normal operation. The DSAM can also be used for electrical machine condition monitoring, machine fault diagnosis, structural characterization and monitoring, among other applications.

## 1. Introduction

The control and reduction of acoustic noise is a wide reaching and public matter which involves health issues and comfort parameters. The levels of acoustic noise must meet certain directives and other regulations, with special rules for public areas and for industrial applications and facilities involving machinery. Among other applications, the monitoring of vibrations in electrical machinery serves to control the acoustic noise, for machine condition monitoring to prevent failure, or simply as an analysis and diagnosis tool. The acoustic noise emitted by machinery in general has been subject for research since the early decades of the last century, which regained interest with the introduction of switched reluctance rotational motors (SRM) for variable speed applications. This is in fact the main drawback on the acceptance of switched reluctance drives, as counterpoint to their simplicity of construction, robustness, reliability and high values of force/torque produced.

Some of the sources of vibrations and acoustic noise in switched reluctance drives are different than for ac machines, as they have a single or a doubly salient structure and no windings or magnets on the rotor [1]. Its origin, control and mitigation have been under study from several years and are related to force/torque ripple under normal operation, structural aspects and aerodynamic issues. The methodologies to reduce the vibrations involve new design strategies, and structural and construction issues, namely the number of poles, the pole shape, and different control methodologies to reduce the torque/force ripple.

The vibrations and noise produced by SRM are periodic signals as the movement is rotational. To perform their characterization one may use time or frequency domain analysis employing Fourier tools, namely the Discrete Fourier Transform (DFT). The types of sensors required are essentially accelerometers and microphones and their number is usually small. As the vibrations are associated to the displacement of coupling or moving parts, namely the shaft and the roller bearing, three 1-axis or one 3-axis accelerometers are normally used in the case of SRMs. The collected data contains information on the mechanical vibrations and can be compared and validated with the acoustic noise produced by using a microphone.

Although one can look at the design of linear switched reluctance machines as the linearization of SRM, there are some differences, such as: the phase windings can be either at the stator or at the translator, although typically they are associated to the translator; the movement is linear and normally longitudinal and not periodic; the number of teeth of the moving part depends on the dimensions of the actuator, namely its length. Some characteristics and dimensions of known LSRAs based on the 6/4 SRM are:

- (a) 6 poles on primary; 1.80 m secondary length; force produced 98.6 N at 5 A phase current [2];
- (b) 120 poles on primary; 4.80 m secondary length; force produced 50 N at 9 A phase current [3];
- (c) 6 poles on primary; 1.90 m secondary length; force produced 100 N at 3 A phase current [4].

In the one hand, the literature covering LSRAs is sparse and only a few known works deal with the vibration problem. Therefore, with reference to SRM, this is a research area that is somehow at the initial stage. Besides the loss of periodicity of SRMs, the vibrations produced by LSRAs depend on the force profiles and structural aspects and on the position of moving parts of the machines. Moreover, the finite length of the machine has to be considered in the propagation of the mechanical waves.

**Figure 1.**
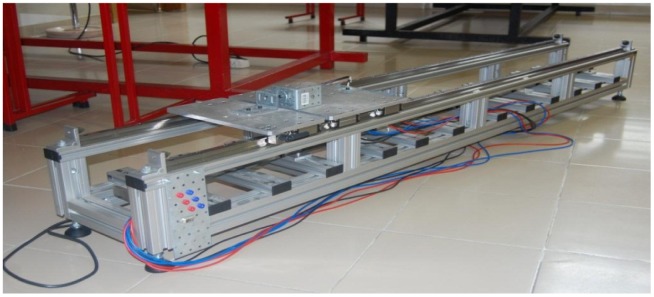
Overview of a LSRA for high precision positioning applications.

The LSRA shown in Figure 1 was designed for high precision applications [4] and serves as the object of study for LSRA characterization in terms of vibrations and noise produced. It has three phase windings at the translator, a length of 2.0 m, 0.5 m depth and is mainly built with aluminium frame profiles, except for the secondary and other magnetic circuit parts, which are of ferromagnetic steel, and the knobs and feet (plastic and rubber).

**Figure 2.**
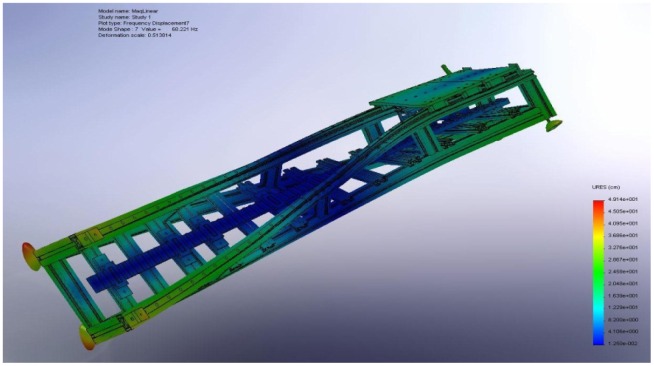
View of the mode shape 7 (74.8 Hz) resultant from the LSRA finite elements simulation [5].

A previous study on finite elements analysis for this LSRA [5] evidences the frequencies of vibration and the mode shapes, whose deformation is illustrated in Figure 2. The displacement of the parts varies with the position, with structural and assembly issues. As a consequence a large number of sensors are needed for the characterization of LSRA, and localized analysis tools are required for time‐frequency or space‐frequency analysis, such as the discrete wavelet transform (DWT) [6,7].

## 2. Overall System Architecture

**Figure 3.**
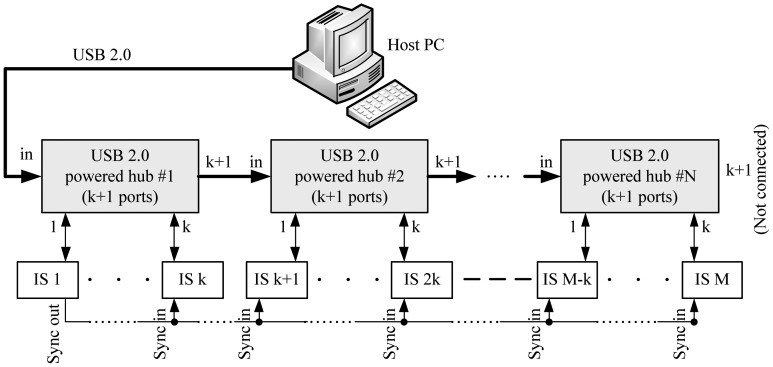
Proposed system architecture: general overview.

To characterize the LSRA in terms of vibration and acoustic noise produced in normal operation, a DSAM was developed, based on intelligent sensor (IS) modules, which are connected to accelerometers and placed in different mechanical parts along the structure of the LSRA. A host computer connected to all IS modules provides user interface, performs system supervision, data collection and signal analysis and representation. The general architecture of the DSAM is shown in Figure 3.

The IS modules connect to the host PC via a USB 2.0 communication channel which also provides the supply voltage (3.3 V) for each IS module. Considering the large number of IS modules and the amount of data for transmission the USB 2.0 protocol was chosen to connect the host PC and the hubs, with a high speed data transmission rate of up to 480 Mbps. In theory it allows the connection of up to 127 high speed (480 Mbps) modules grouped and connected to USB 2.0 with external power.

The system operates with selectable sampling frequencies in submultiples of 160 kHz (the default sampling frequency) and using asynchronous or synchronous data acquisition modes. For the synchronous mode operation, an external 2-wire line (Sync and GND) connects any IS module (by default is the first one in the chain) to the remaining IS modules, thus allowing for a faster and more flexible synchronism feature. The host PC controls the all system through a Matlab^®^ (Matlab is a registered trade mark of The MathWorks Inc.) application also developed by the authors.

### 2.1. IS Module Architecture

**Figure 4.**
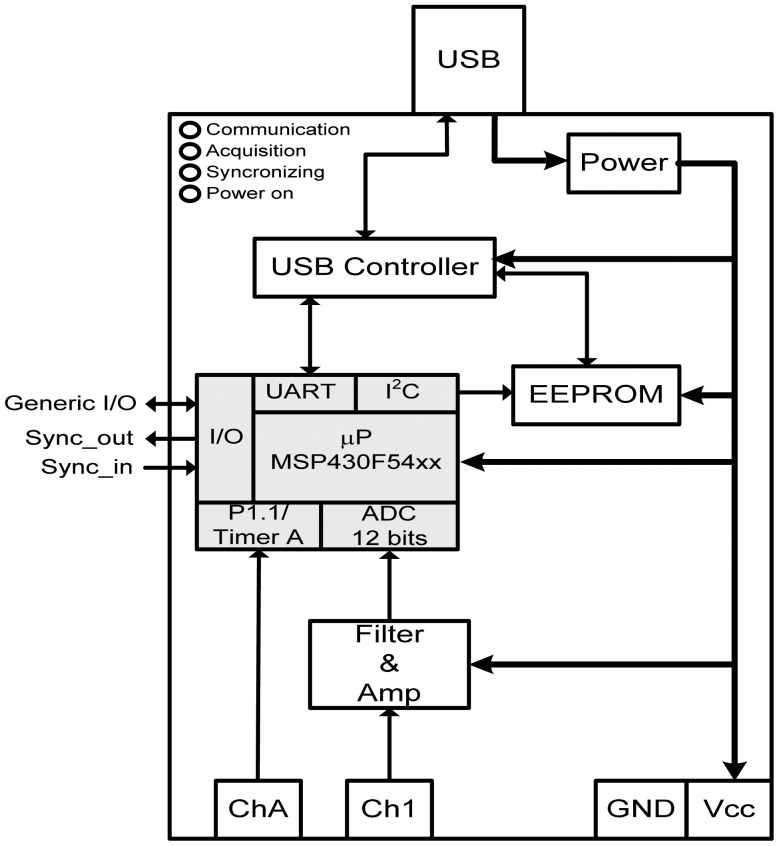
Diagram of the IS module architecture.

The IS module, whose functional diagram is depicted in Figure 4, is based on the MSP430F54xx low power microcontrollers (MCU) manufactured by Texas Instruments [8]; it has a communications interface block and two channels for sensor/transducer connection. The IS module allows the connection of different type of sensors/transducers, although not simultaneously: with analog output or digital serialized frame output. This feature provides system flexibility on the use of different type of sensors/transducers and justifies the existence of Ch 1 (for analog output devices) and Ch A inputs for PWM output devices. Associated to Ch 1 there is an analog anti-aliasing filter.

The length of memory reserved for data acquisition (data buffer) is 15 kByte. The MSP430F54xx internal ADC is of SAR type with 12-bits resolution, thus data can be stored either as 8- or 16-bit samples. However, the latter option restricts the effective sampling rate to half, *i.e.*, for a frame corresponding to 1 second, the sampling rate is 15 ksamples/s for 8‐bit data, and 7.5 ksamples/s for 16‐bit data. The sampling process ends when the memory data buffer is full and the IS enters in an idle state waiting for data collection from the host PC. After that, a new sampling and data acquisition cycle may start. The data acquisition depends on the configuration of the IS trigger which can be internal or external. For the internal trigger situation, the data acquisition starts immediately after the reception of the sampling command, sent by the PC, and as a consequence the Sync_out line goes to the high level. For the external trigger case, the acquisition starts when a low-to-high transition occurs at the line Sync_in, *i.e.*, it is edge-triggered. This allows that one IS can be used to synchronize all the other IS during data acquisition.

To reduce the latency to a minimum, the Sync_out port of the “master” IS module connects in parallel to the Sync_in port of the other IS modules. Thus, the delay on acquisition time among the first and the other IS modules corresponds to the time of processing the instruction for acquisition. The time delay measured for Sync signal is around 2.4 μs, which is better than the worst case sampling period, which is 6.25 μs (@ *f_S_* = 160 kHz). This guarantees that there is no loss of simultaneous samples and, for the worst case, the loss is one sample at the leading IS module with reference to the others. This feature allows the correlation of data acquired by the different IS modules.

The communications interface between the UART of the microcontroller and the host PC is performed by the USB controller. The TUSB3410 device from Texas Instruments is used as USB controller [9] and the EEPROM contains the necessary firmware for the initialization of the USB controller. The UART embedded in the TUSB3410 allows a baud rate ranging from 50 to 921.6 kbaud, selectable by software. For 8-bit per symbol, with start and stop bits, the communication speed does not exceed 10 Mbps, which corresponds to a full speed USB communication channel (10 Mbps max speed). In one hand, the high speed communication rate implemented by the USB 2.0 hubs is equivalent to 48 full speed USB modules. However, for the majority of applications, the number of expected IS modules is much lower and shall not exceed 32, even for larger LSRA monitoring, thus the bandwidth available is sufficient for this application.

### 2.2. Communications Management

Table 1 summarizes the structure of commands, both from the host PC and the IS module. After start up, the IS module waits a command from the host PC within one of the following possible identifiers: C: Configuration; D: Data.

The D command requires only two bytes: the first corresponds to the command identifier and the second contains the IS identifier. By its turn, the configuration command needs a 5-byte array: the first is the command identifier; the second contains information on the ADC resolution; the third and fourth bytes allow adjusting the sampling frequency (define the number of clock periods correspondent to a sampling period) [10] and the last one is the IS identifier.

### 2.3. Firmware Organization

The host PC configures the IS module and it acknowledges a successful configuration by sending the string ‘OK’. Upon success the configuration is set in order that the line Sync_out goes to the high level. The acquisition process starts when executing a data collection command or when an edge transition occurs on the line Sync_in. The latter corresponds to the synchronous operating mode: the Sync_out line of the “master” IS module is connected to Sync_in of the other IS modules.

**Figure 5.**
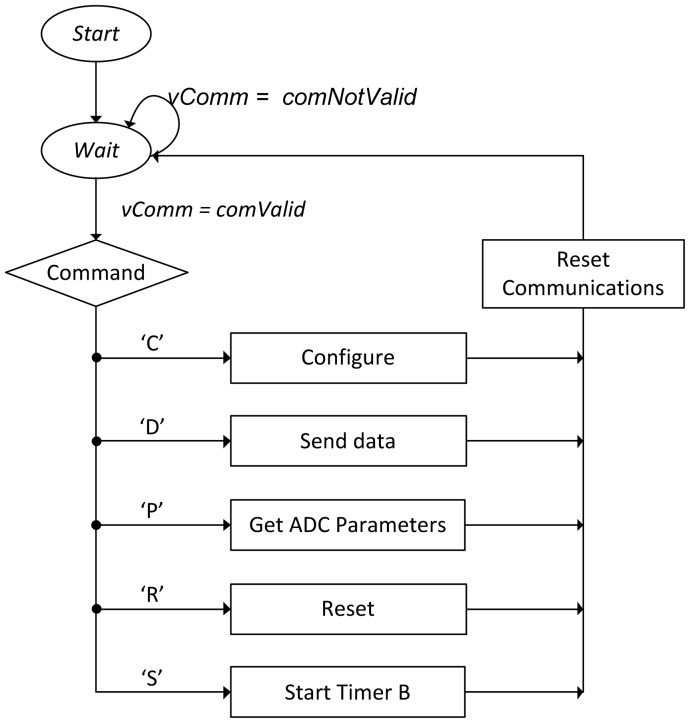
General flowchart of the firmware operation.

The acquisition is controlled by TIMER_B of the MCU which must be duly configured according to the desired sampling frequency, in order perform a complete acquisition and store data in the data buffer. The flowchart of Figure 5 describes the operation of the firmware for communications.

**Figure 6.**
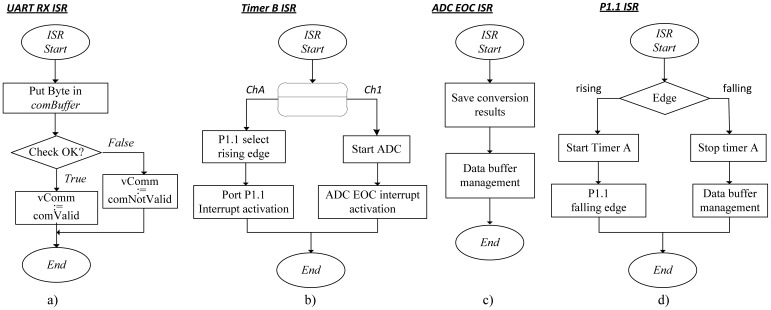
Flowchart of the service routines: (**a**) serial communications; (**b**) data conversion; (**c,d**) Data store and buffer management.

The internal operation of the IS modules uses four interrupted routines. The interaction of these routines, depicted in Figure 6, allows the correct execution of the IS module and the desired functionalities.

The service routine for communications UART_RX_ISR starts always with the reception of one byte sent by the host PC. Its function is to collect the byte from the reception register, RX, and move it to the communications buffer, CommBuffer. At the end, upon success, the data packet is validated and a signaling LED is activated.

The TIMER_B_ISR routine is activated with the beginning of the acquisition process. This routine is executed with a fixed periodic rate, whose periodicity is established during configuration, and according to the sampling period. This routine activates the right resources depending on the channel used for acquisition (Ch1 or Ch A). If Ch 1 is used it starts the analog-to-digital conversion process and activates the End-of-Conversion interrupt. The result of the ADC conversion is collected by the ADC_EOC_ISR which stores it in the data buffer. On the other hand, if Ch A is used, an interrupt at port P1.1 is activated for a low-to-high edge transition. Finally, the interrupt service routine P1_1_ISR has two possible operations. For a positive edge transition, Timer A is activated and stopped for a negative edge transition. The management of the data buffer is required for both cases. When reaching the end of the memory allocated for the buffer is reached the acquisition process is disabled and Timer B counting operation is inhibited.

### 2.4. Sensors and Sensor Interfaces

**Figure 7.**
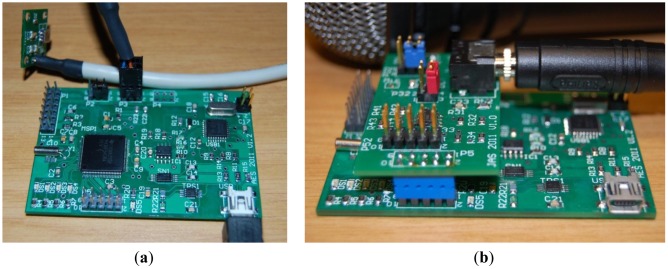
(**a**) Base configuration of the IS module with the connected analog output accelerometer board; (**b**) IS module configuration with an audio interface board fitted and a connected 3.5 mm audio jack (the microphone is partially visible in the background).

To achieve its main purpose—the analysis and characterization of vibrations and acoustic noise in linear switched reluctance actuators—the IS modules have a base configuration allowing the connection of analog or digital output accelerometers but also an add-on interface audio board that fits into the sensor module to monitor the audible noise, as shown in Figure 7(a,b). The add-on interface board has an in-board omnidirectional microphone (mounted on the bottom face) and a 3.5 mm audio jack to connect an external microphone. Both lines are fed to an audio amplifier whose output is connected to the analog input (or Ch1) of the IS module.

In the current version, data from vibrations are collected by state-of-the art MEMS 1-axis accelerometers with ±70 g sensitivity, and 20 kHz bandwidth [11]. These sensors have analog output, a sensitivity of 16 mV/g (typical), good linearity (0.2% full scale non-linearity), low noise ( $4\;\text{mg}/\sqrt{\text{Hz}}$) and frequency response up to 22 kHz, thus covering the audio band. The 0 g value corresponds to an output voltage, *V_S_*/2, *i.e.*, 1.65 V for a 3.3 V supply voltage.

The frequency response of the accelerometers denotes a 0 dB level flat response and a ∼6 dB resonance peak at ∼20 kHz. For this reason the RC filter sections at the accelerometer output and the anti-aliasing filters at the analog input channel (Ch1) are designed to have a cutoff frequency (−3 dB) at 13.8 kHz with −5 dB at 20 kHz. This allows one to obtain a nearly 0 dB magnitude level for the all audio band without the need for signal conditioning amplifiers. The IS modules with the audio interface board fitted have section filters designed for a cutoff frequency (−3 dB) at 8 kHz, taking into account the technical characteristics of the microphone but also the sensitivity of the common human ear. The connection of current sensor modules [12] with analog output voltage is also allowed for complete characterization of the LSRA in terms of vibrations produced according to the current profiles in the coils.

### 2.5. Software Application and Signal Analysis Tools

**Figure 8.**
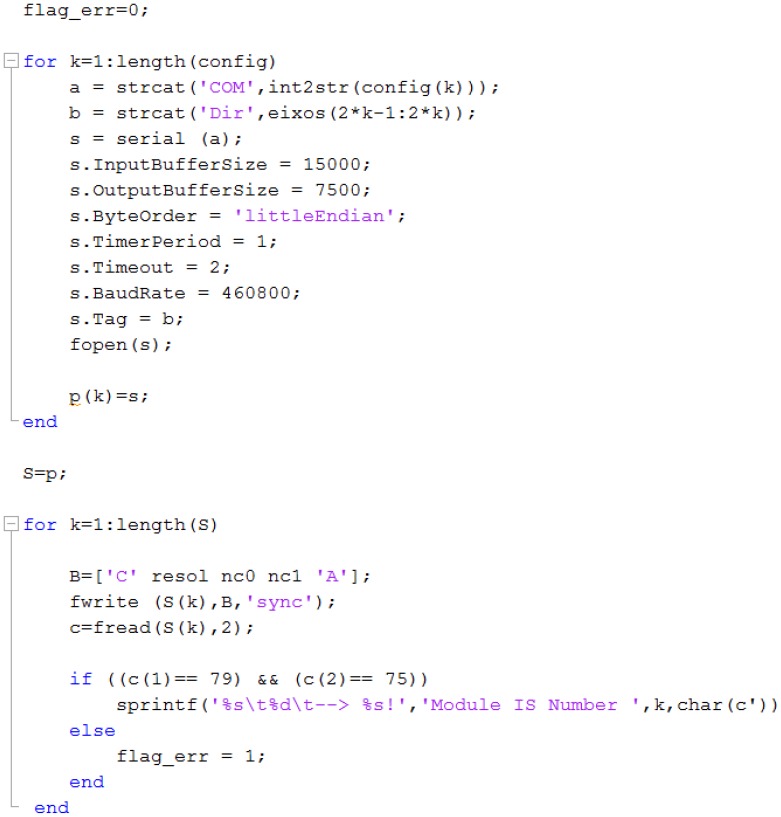
Excerpt of code for the system configuration and IS modules registration.

Figure 8 shows part of the code related to the first software block, beeing visible the commands for setting up the communications with all the IS modules registered in the host PC operating system.

**Figure 9.**
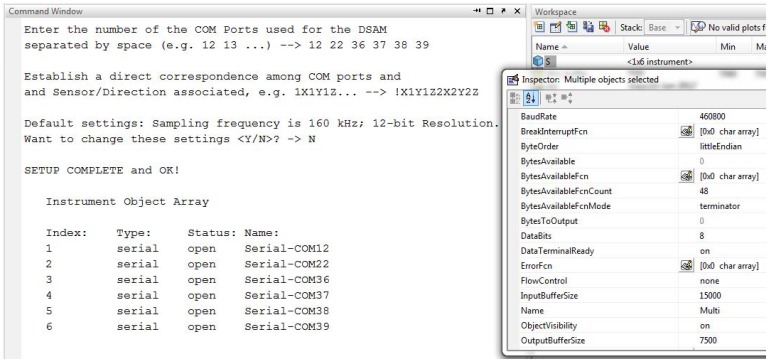
Diagram of the IS module architecture.

The software application, developed in Matlab, is modular and composed by three functional parts: (1) system configuration and setup; (2) system management, data acquisition and data storage (data collected is stored in *.mat files); and (3) for data and signal analysis and representation. Upon success the IS modules become registered in Matlab as external instrument objects, as depicted in Figure 9, and its properties can be inspected by opening its object identifier.

The other two software modules include functions and commands for data acquisition, Matlab built-in functions, namely for serial I/O, and other functions developed by the authors for signal analysis and representation. At the current version the Discrete Fourier Transform (DFT) is used to estimate the signal spectra but other tools may also be considered. For practical spectrum analysis, a tapered window is often used. For vibrations on the audio range of frequencies the Blackman/Blackman–Harris windows are commonly used but other window functions, such as Hamming, Hanning, Bartlet and Parzen can also be considered also as other analysis tools [6,7].

## 3. Some Experimental Results and Example Applications

Several tests were conducted to evaluate the system's functionalities, performance and reliability, namely to evaluate the robustness to connectivity and communication, and to evaluate the main features, *i.e.*, data collection and signal analysis, whose results are present and discussed in the following sections.

### 3.1. Connectivity and Communication Stability and Robustness

At the current development stage, the system can accommodate up to 28 IS modules grouped in four 7-port USB 2.0 hubs. This configuration is considered satisfactory to the requirements as for the majority of applications, regarding the main purpose of the system, the expected number of IS modules to use simultaneously shall not exceed 23, according to the following configuration: 18 for 1-axis accelerometers; two for audio data collection and three for the coil current measurements.

Since the number of USB devices connected and registered simultaneously on the operating system is above that seen in most common applications, one needs to ensure the system robustness, stability and reliability for communications. The operating system may denote some instability during the process of identifying USB devices, and may take some time to successfully identify them, especially for a large number of IS modules. On the one hand one must expect stability after device registration. On the other hand some problems may arise if the IS modules are powered via the USB ports, although the devices used, namely the microcontroller have low-power consumption characteristics.

The device registration and stability are features exclusive dependant on the host PC characteristics, on its operating system and the external hardware used for communication. Therefore they are not considered as intrinsic characteristics of the DSAM and therefore susceptible for evaluation. However they can compromise significantly the system performance and functionality: if a USB connection broke or failed but the associated IS module is registered in the Matlab application and a COM port is allocated to it, then a system failure occurs. Therefore, the evaluation considers the connection stability and robustness of the communications, to ensure the proper system operation. The communications using 4-port unpowered USB 2.0 hubs and 10-port uncertified USB 2.0 hubs with external power were evaluated previously. In both cases the tests revealed instability after connecting the forth IS module. To overcome any Operating System (OS) instability, certified USB 2.0 hubs are used for evaluation, either for the 4-port or 7-port hub units, all from the same manufacturer and the latter with an external power unit.

**Figure 10.**
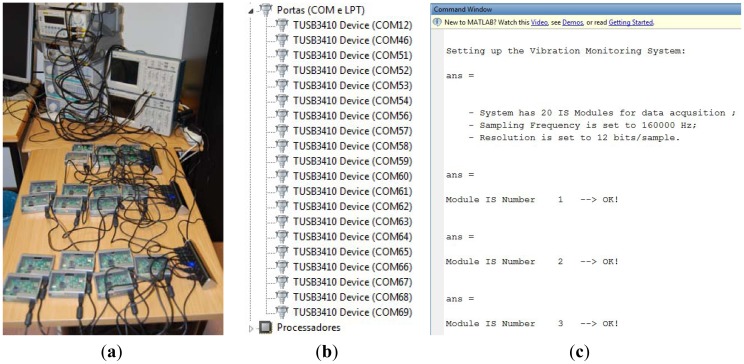
(**a**) General overview for 20 IS modules connected to three USB hubs; (**b**) COM ports registered at the host PC operating system; (**c**) Messages at Matlab prompt window.

Figure 10 shows a general overview of the distributed system with 20 IS modules connected to three 7-port USB 2.0 hubs, the COM Ports registered in Windows 7 OS and the messages shown at the Matlab command window at system setup and configuration.

At its maximum capacity, with four 7-port USB 2.0 hubs with external power, which by its turn are connected to a 4-port USB 2.0 hub, the system can accommodate up to 28 IS modules. This satisfies the maximum expectations of IS modules while keeping five spare ports. Normally the UART embedded in the TUS3410 controller is configured by software to communicate at a half of its maximum baud rate, *i.e.*, 460.8 kbaud. As a consequence the IS modules communicate via a USB link at a speed around 5 Mbps. As the USB 2.0 hubs manage the bandwidth according to the effective communication needs of the controller devices connected to it, the effective overall speed of the system, using 28 IS modules, is around 140 Mbps. The effective overall speed is nearly 280 Mbps if the UART is configured to its maximum baud rate.

In Figure 10, the length of the USB cables connecting the IS modules to the hub is around 0.8 m and the length of the cable connecting the 7-port USB hub to the 4-port hub is around 1.6 m. The 4‐port hub fits directly into the host PC (its cable length is less than 0.10 m). The system showed stability. Another test was carried out using an extra cable with 3 m to connect the host PC to the 4-port USB hub and connecting the 7-port USB hubs to its USB plugs, although using only 6 IS modules were used. In both tests the communications were stable and reliable.

The total cable length achieved in this later situation is around 5.5 m, *i.e.*, with a 10% extra length over the maximum admissible cable length for high speed of full speed USB connections, which is 5 m, regarding a maximum propagation delay of 26 ns, and the attenuation of the twisted pair for the differential data lines (minimum is a 28 AWG twisted pair cable). The cable length depends on the characteristics of the cable used, namely its capacity and the associated propagation delay, which must be less than 26 ns with operating frequencies up to 480 MHz. For high quality cables the propagation delay is around 6.5 to 7.0 ns/m thus leading to a maximum cable length of 4.6 to 4.3 m, respectively [13]. Based on these results it is expected that the system keep its functionalities and stability for average cable lengths around 4.5 m in total, which is the maximum total cable length expected for communications at the end user application.

### 3.2. Data Collection and Signal Analysis for Standard Waveforms

The IS modules were all previously tested independently, using worst-case signals, in order to find any possible malfunction, non-linearities, device limitations or susceptibility to noise. In order to minimize the influence of noise, clip-on ferrites were added to the USB cable, at the end connecting to the IS module via mini-USB connector. All IS modules performed as expected, according to the resolution of the ADC and its linearity parameters. The tests considered the limits of frequency and the limits of the dynamic range, especially for the lower limit of magnitude.

To evaluate the functionalities for data collection and signal analysis, six IS modules were connected to a USB hub and standard waveforms signals from six different signal generators were applied directly to each one. In order to have references, the waveforms were also displayed on digital storage oscilloscopes with on-screen measure functionalities.

The IS modules were configured for synchronous data operation with a 160 kHz sampling frequency and a resolution of 12 bit/sample. Low amplitude values signals were applied in order to evaluate the sensitivity of the system, in accordance with the sensitivity of the majority of the accelerometers used.

**Figure 11.**
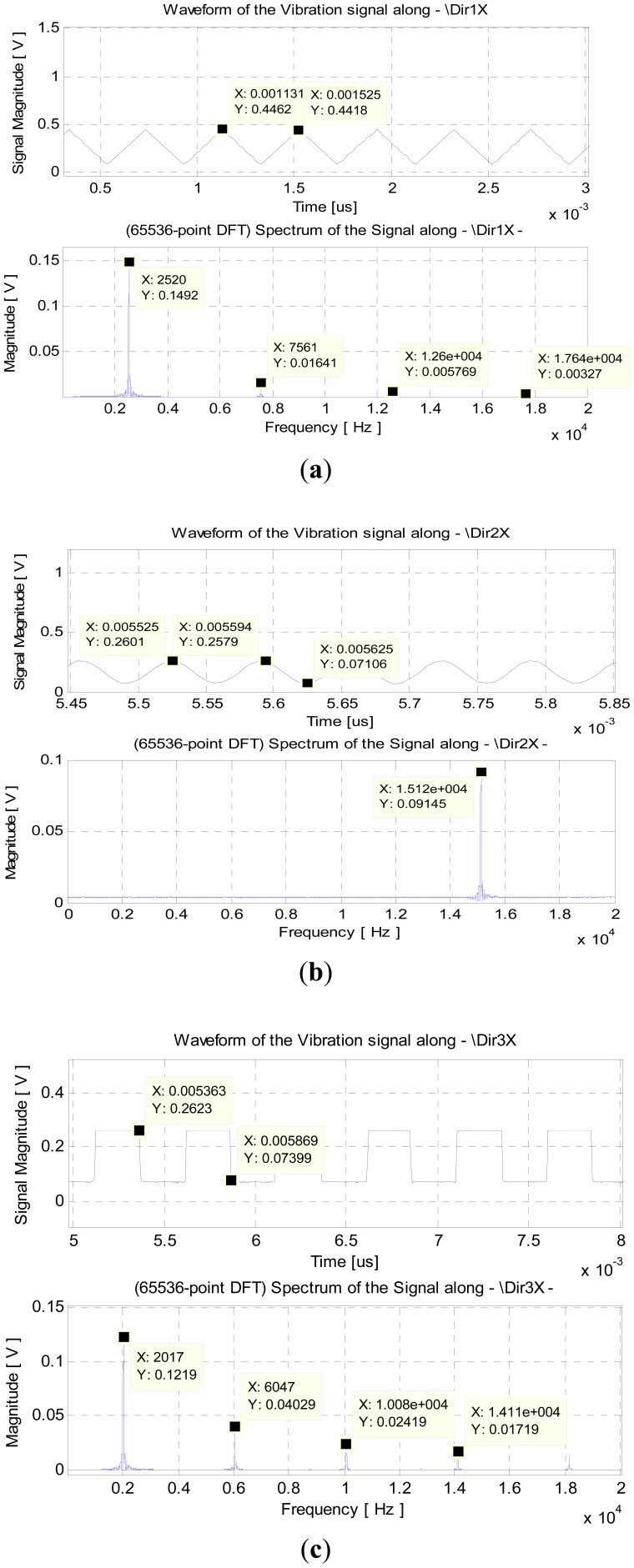
(**a**) Triangular waveform; (**b**) Sinewave; (**c**) Square wave.

To illustrate the results obtained, only the signals from the first three IS modules are shown, whose input signals were, respectively: a triangular waveform with 0.4 Vpp, 0.25 V offset and frequency 2.5 kHz; a sinusoidal signal with amplitude 0.2 V, offset 0.15 Vdc and frequency 15 kHz, and a square wave with 0.2 Vpp amplitude, 0.15 Vdc offset and frequency 2 kHz. The results obtained for the discrete-time and frequency domains are presented in Figure 11.

As shown in Figure 11, in the discrete time domain representation, the magnitude is ∼370 mVpp and the period is ∼395 μs, which corresponds to a frequency of ∼2.53 kHz. In the frequency domain, for the fundamental frequency, the amplitude is ∼150 mV and the frequency is 2.52 kHz. These values are similar and coherent with those of the continuous time signal. Moreover, the spectrum shows the harmonics contents associated to the odd-ordered harmonics: the third, the fifth and seventh order harmonics are at 7.56 kHz, 12.6 kHz and ∼17.6 kHz, respectively. This result is typical for this type of waveform and is similar to its analysis with discrete Fourier series (DFS): the coefficients of DFT and DFS are coincident. On the other hand, the energy associated to the fundamental frequency is spread, thus causing the loss in magnitude at the fundamental frequency. In a similar way, in the second case one obtains a sinusoidal waveform with magnitude ∼95 mV and the period is around 66–67 μs, thus the frequency is approximately 15.1 kHz, which is also coherent and in-line with the values obtained at the frequency domain, and those considered for the original signal. In this case, due to the absence of harmonics, the energy of the signal is associated to the unique frequency, and therefore the frequency remains almost equal. Finally, in the third case, a square wave signal is obtained with magnitude ∼190 mVpp and period ∼505 μs and the corresponding frequency is ∼1.98 kHz. The results obtained for the spectral representation are once again coherent: the frequency is ∼2.02 kHz and the magnitude for the fundamental frequency is ∼130 mV. In this case there is also odd-ordered harmonics which cause a loss on amplitude at the fundamental frequency. With reference to a digital oscilloscope, for the same signals, the values measured for frequency have an error (deviation) less than 1%. The values measured for amplitude have an average error of 5% and maximum of ∼8%.

### 3.3. Monitoring the Vibrations of a Single-Point Supported Beam

**Figure 12.**
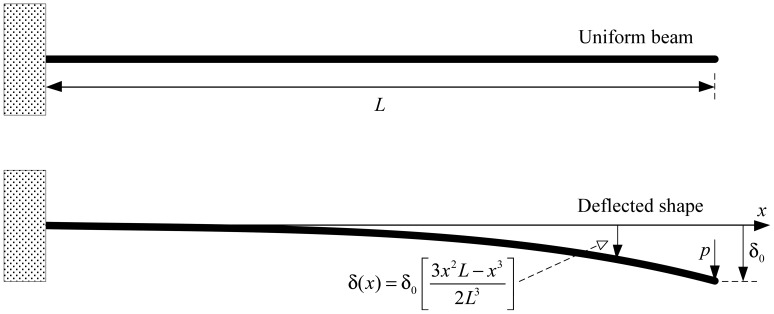
Simplified analysis of vibrations for a SDOF deflected beam.

Experimental tests to monitor the vibrations of a single point supported beam were also performed to validate the system's operation using non standard, non-deterministic, low voltage signals obtained from the accelerometers' output. For simplicity a uniform beam is considered, assuming that the displacement only occurs along the y-axis direction; thus it is a single degree of freedom (SDOF) mechanical system [14]. Due to the action of the gravity the beam tends to deflect and assume a curved shape, as shown in Figure 12.

As the beam is only subject to the force of gravity, using d'Alembert's principle, the equation of motion for the system is:

(1) $$m\ddot{\upsilon }\left(t\right)+c\dot{\upsilon }\left(t\right)+k\upsilon \left(t\right)=p(t)$$

In Equation (1) *m* is the mass, *c* the damping coefficient and *k* the stiffness, *υ*(t) the displacement of the mass with respect to a reference initial point when *p*(*t*) = 0, *υ̇*(*t*) it the velocity and *ϋ*(*t*) the acceleration. Equation (1) states that the sum of all forces acting in a mass *m* should be equal to zero with *p*(*t*) an external force (gravity), *mϋ*(*t*) the inertial force, *cυ̇*(*t*) the damping force and k*υ̇*(*t*) the restoring force. The solution to Equation (1) is obtained for its homogeneous form, *i.e.*, when its right hand side is set equal to zero. The method is a simple approach to calculate the resulting cantilever deflection and the displacement at the end and at various points (positions) along the deflected beam. Considering the displacement at the end of the beam linear and small with respect to the free length, one can compute the first mode by using the Rayleigh method with cubic approximation for interpolation functions, in the form:

(2) $$v\left(y\right)=ay^{3}+by^{2}+cy+d$$

The displacement at the end of the beam shown in Figure 12 is calculated by:

(3) $$\delta_{0}=\frac{p\cdot L^{3}}{3E\cdot I}$$

The deflection shape for other positions along the beam is given by:

(4) $$\delta \left(x\right)=\delta_{0}\left(\frac{3x}{2L^{2}}-\frac{x^{3}}{2L^{3}}\right)$$

Using the Rayleigh method, the vibration frequency of the first mode is defined by:

(5) $$f=\frac{1}{2\pi }\sqrt{\frac{140}{11}\cdot \frac{E\cdot I}{\bar{m}\cdot L^{4}}}$$

In Equation (5)*E* is the Young's modulus, *I* is the inertial momentum, the product *E I* is the stiffness, *m̄* is the distributed mass and *L* is the length.

**Figure 13.**
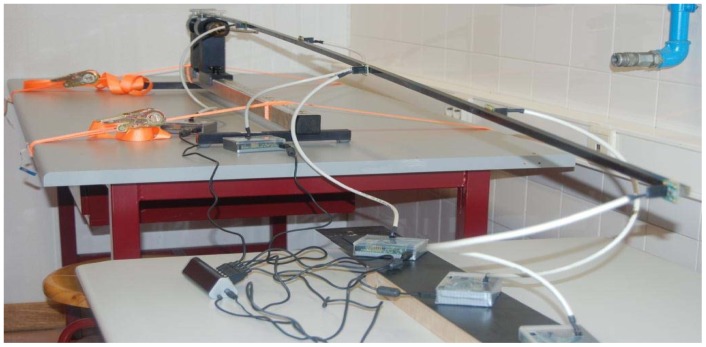
Test bench for the deflected beam: accelerometers and IS modules are visible.

To evaluate experimentally the model of Figure 12 the test setup of Figure 13 was implemented. The experimental procedure consists of forcing the free end on the beam to its horizontal position, and then leaving it to the action of the gravity, thus starting a free dumped oscillation regime. A beam of steel with 10 × 10 mm section and length 2.0 m is mounted and duly fastened with screws to a mechanical support in one end, leaving a length of 1.9 m for free displacement. The mechanical support is tied to a bench, and the bench is fastened with screws to the floor, ensuring no other oscillations. The bench is set to the level and the beam is aligned with the central longitudinal axis on the base of the mechanical support. Finally, five accelerometers were attached to the beam using bee-wax, regularly separated by 0.4 m along its length, starting from its free end. With reference to the fix end, the positions with accelerometers are: P_5_ (0.3 m); P_4_ (0.7 m); P_3_ (1.1 m); P_2_ (1.5 m); P_1_ (1.9 m).

The values of Young's modulus and the mass of the beam were obtained experimentally: *E* = 213 × 10^6^ Pa and *m* = 1.598 Kg. The initial deflection, measured at the end of the beam with respect to its horizontal position (determined through a scale and spirit level tools) is around 0.11 m. Using these values in Equation (5) one obtains a frequency ∼3 Hz for the first vibration mode. Numerical simulations to calculate the first 7 modes, its frequencies, the mode shapes of the mechanical wave and the associated displacements were also performed. Table 2 resumes the first three vibration modes where δpk is the peak displacement of the mechanical wave, Lpk is the position for which the peak occurs (referenced to the fixed end), and $S_{k}^{*}$ refers the sensors at position *k* excited by the mode.

**Figure 14.**
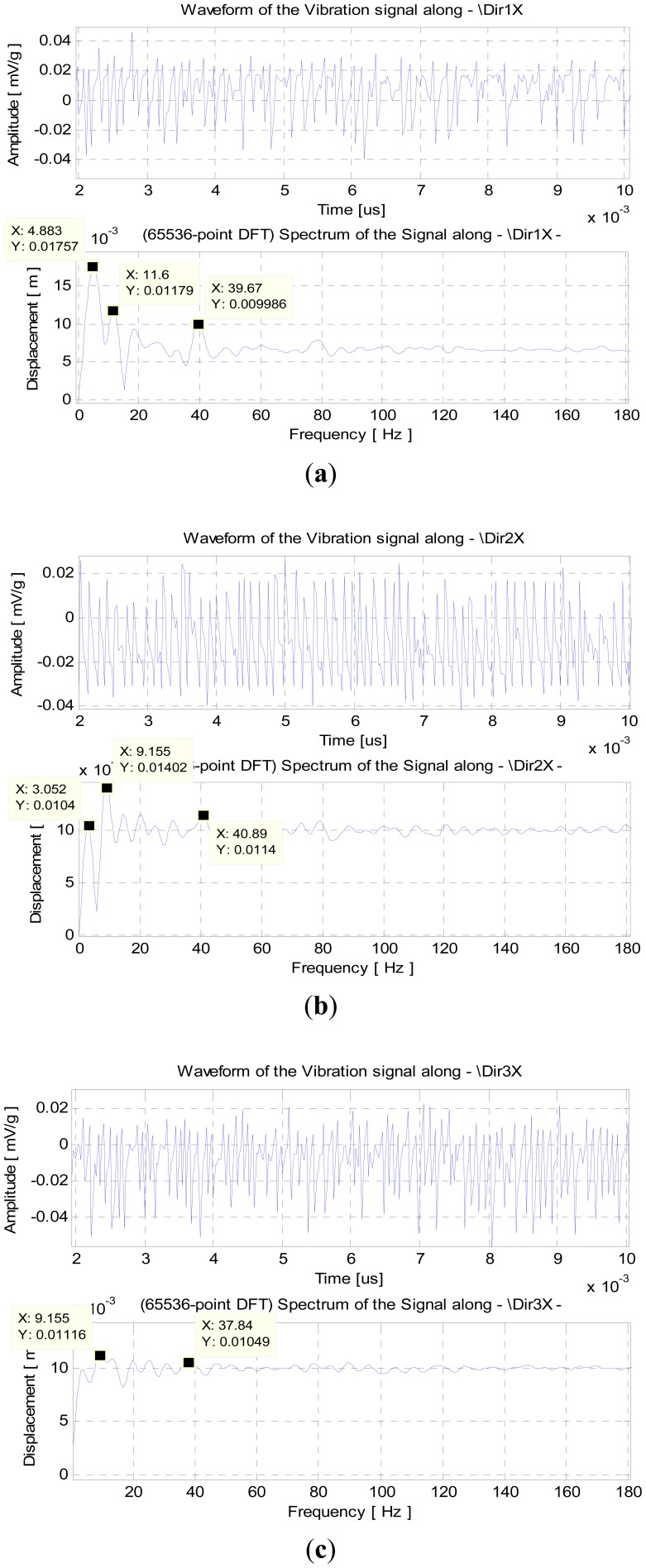
(**a**) Signal at sensor S_1_; (**b**) Signal at S_2_; (**c**) Signal at position P_3_ (S_3_).

The experimental data is collected for several points along the beam in synchronous operation mode. To minimize unbalance due to the inertia of the sensors and cables connecting to the IS modules, and its effects in oscillations, the sensors were attached in an interleaved scheme, in both sides of the beam, as shown in Figure 13. The experimental results obtained for the damped oscillations are shown in Figure 14.

Figure 14(a) shows the results of the spectral analysis of the signal obtained from the sensor S_1_ at position P_1_. The vibration frequencies associated to the first three vibration modes are visible: ∼5 Hz to mode 1, ∼12 Hz to mode 2 and ∼40 Hz to mode 3. With the exception of the first mode, the experimental values are similar to those obtained by numerical simulation. The analysis of the signal taken from sensor S_2_ evidences mainly the frequencies of modes 2 and 3, its values are similar even though the frequency of mode 2 suffers some variation. The frequency of the first mode is also noticeable with a very close value to the expected one with reference to the simulations. Finally, the spectral analysis of signal at sensor S_3_ shows mainly the frequencies of mode 2 and 3, although mode 1 is also present but with less amplitude, and thus with less significance.

The frequencies' deviations, especially for mode 1 at sensor S_1_, are due to non ideal conditions. In one hand, the real beam is not uniform as its section dimensions vary along its length—the average values are 10.1 × 10.2 mm; the inertia of the instruments and cables influences the motion of the beam; at the initial position the central point of the beam's section must be aligned with the longitudinal axis. All these conditions tend to influence the beam towards a circular or elliptic motion rather than linear. These factors suggest the influence of other modes excited by the torsional forces resulting from the circular or elliptic motion, which are not considered in the linear motion SDOF model of Figure 12. Moreover, according to Table 2, the signal at S_1_ (position 1.9 m) is influenced by modes 1, 2 and 3, which means that the approximation by the Rayleigh method is too simple. As a consequence, one needs to consider multiple degrees of freedom systems and use superposition analysis methods, with an increase on the complexity.

Several test sequences were performed and the results obtained showed very small differences with those obtained from simulation, thus confirming system's repeatability. The results obtained experimentally for worst case low valued standard waveforms, and for non-deterministic vibration signals are in accordance to those obtained from numerical simulations, although with some minor differences. The experimental results support the system's repeatability, reliability, and accuracy within the error margin of the ADC, namely the linearity error parameters, which typically are less than ±2 LSB.

Although the model considered in Figure 12 is very simple and related to a SDOF mechanical system, it allows demonstrating the system's functionality and reliability. The very same principles can be applied to experimental modal analysis of more complex mechanical systems, with multiple degrees of freedom (MDOF), such as the LSRA, by representing the system transfer function by using matrix notation or applying linear superposition, considering several SDOF models. Therefore, the DSAM can be deployed both for experimental modal analysis and to collect data from the vibrations and the acoustic noise produced under operation, aiming the full characterization of a LSRA.

## 4. Conclusions

This paper proposes a distributed system for monitoring and analysis of vibrations aiming the characterization of linear switched reluctance actuators in terms of acoustic noise produced. The system architecture is presented, and its features are described. The advantages of the proposed system are its modularity, scalability, and flexibility and the possibility of using different types of sensors, either analog or digital outputs, and for signals of different nature. The cost is also a strong point, as it is lower than that of any other available commercial solution. The DSAM is reconfigurable, can operate either in synchronous or asynchronous modes, programmable sampling frequencies, 8-bit or 12-bit resolution, and a memory buffer with 15 kbyte length.

The experimental results demonstrate the robustness of the connection and communications for the USB 2.0 protocol under the Windows 7 operating system. The effective communication speed for each IS module is configurable by software and never exceeds 10 Mbps. At this maximum speed, by using high speed USB 2.0 hubs (max 480 Mbps) the DSAM allows the connection of up to 48 IS modules, which is considered sufficient regarding the number of sensors needed for an LSRA characterization.

Although with some differences with the numerical simulated references, mainly due to the simplicity of the model, none of the results invalidates or diminishes the features of the proposed system. Rather, the results obtained experimentally for standard waveform signals, and for low value vibration signals obtained from accelerometers demonstrate that the proposed system is well suited for its main purpose: to support the analysis and acoustic noise characterization of linear switched reluctance actuators, or other applications that require a large number of sensors, also from different types, placed along a structure. The proposed DSAM allows the collection of experimental data to validate the results obtained from finite elements simulation of a LSRA; to have information on structural adjustments to the mechanical model, in order to reduce vibrations and the acoustic noise; to have reliable information on the parts and materials submitted to more stress and therefore to prevent fatigue and failure.

## Figures and Tables

**Table 1.** Communications summary: data packets and commands.

**Command**	**Host PC**	**IS module**
**C**	8 or 16-bit data Ch A or Ch 1 IS module identifier	OK or Not OK
**S**	–	OK or Not OK
**D**	*fwrite fread*	OK or Not OK (15 kB)
**R**	–	OK or Not OK
**P**	–	ADC configuration information

**Table 2.** Characteristics and parameters associated to the first three vibration modes.

**# Mode**	**Frequency [Hz]**	**|δpk| [m]**	**Lpk [m]**	$S_{k}^{*}$
1	∼3	>0.10	1.9	S_1_
2	∼14	0.002	0.9, 1.9	S_1_, S_3_
3	∼41	0.0003	0.6, 1.3, 1.9	S_1_, S_2_, S_3_, S_4_
